# A Knowledge Graph–Enhanced Question-Answering System for Chinese Patent Medicines in Pediatric Influenza: Development and Evaluation Study

**DOI:** 10.2196/101648

**Published:** 2026-11-13

**Authors:** Haoyuan Li, Guozhen Zhao, Xingyu Zong, Ning Liang, Huizhen Li, Wandi Zhang, Jiahao Lin, Bohan Ma, Jinghan Jia, Yanping Wang, Nannan Shi

**Affiliations:** 1 Institute of Basic Research in Clinical Medicine, China Academy of Chinese Medical Sciences, No.16, Nanxiaojie, Dongzhimen Nei Street, Dongcheng District, Beijing, China, 86 010-64093251; 2 School of Acupuncture-Moxibustion and Tuina, Beijing University of Chinese Medicine, Beijing, China

**Keywords:** knowledge graph, large language model, retrieval-augmented generation, pediatric influenza, Chinese patent medicine, evidence provenance, clinical decision support

## Abstract

**Background:**

Pediatric influenza is an important public health concern. Chinese patent medicines are used in its treatment, but relevant clinical evidence is dispersed across guidelines, expert consensus statements, and clinical studies. The multidimensional knowledge required for syndrome-based prescribing complicates information retrieval and standardized clinical use.

**Objective:**

This study aimed to develop a traceable knowledge graph of Chinese patent medicines for pediatric influenza, integrate it with a large language model (LLM) to support evidence-based question answering, and evaluate answer quality and preliminary usability.

**Methods:**

We systematically searched for clinical practice guidelines, expert consensus statements, and clinical studies. Evidence segmentation, ontology development, LLM-assisted extraction with manual verification, terminology normalization, and knowledge integration were used to construct a Neo4j knowledge graph. Structured graph retrieval was combined with retrieval-augmented generation to develop a question-answering system. A standalone LLM and the knowledge graph–enhanced LLM (KG-LLM) each answered 80 standardized questions. Three clinical experts independently evaluated anonymized, randomly ordered answers for core task accuracy, completeness, and unsupported factual content. Five physicians participated in an exploratory usability evaluation using the System Usability Scale (SUS), study-specific Likert items, and self-reported evidence-location outcomes.

**Results:**

We included 142 publications and generated 1294 evidence units. The knowledge graph contained 3211 nodes and 19,671 relationships, linking structured knowledge to evidence units and source publications. Across 80 paired questions, KG-LLM achieved higher accuracy scores than the standalone LLM (mean 3.05, SD 1.88 vs mean 2.02, SD 1.54; Holm-adjusted *P*<.001) and higher completeness scores (mean 3.11, SD 1.84 vs mean 2.42, SD 1.62; adjusted *P*=.002). Among 78 pairs with determinate assessments, unsupported factual content was identified in 11 standalone LLM answers (14.1%) and no KG-LLM answers (0/78, 0%; adjusted *P*=.002). The mean SUS score was 96.50 (SD 2.85). Of 5 physicians, 4 (80%) reported locating both the original publication and the corresponding evidence.

**Conclusions:**

The traceable knowledge graph and graph-enhanced question-answering system integrated clinical evidence on Chinese patent medicines for pediatric influenza. KG-LLM achieved higher accuracy and completeness and less unsupported factual content on standardized tasks than the standalone LLM. Preliminary usability findings were favorable, but generalizability, long-term usability, and safety in clinical practice require further evaluation.

## Introduction

### Background

Influenza is an acute respiratory infection caused by influenza viruses. Influenza A viruses can cause global pandemics and pose a continuing threat to public health [1]. Children experience a high burden of influenza and are at increased risk of severe disease. In particular, children younger than 5 years may develop serious complications or die after infection. Chinese patent medicines have been reported to have therapeutic advantages, including antiviral, antipyretic, analgesic, and immunomodulatory effects, relatively few adverse reactions, and a low propensity for drug resistance. They have, therefore, become an important treatment option for pediatric influenza [2]. Several guidelines and consensus statements have been published to standardize their use, including the Guidelines for Clinical Application of Chinese Patent Medicines in the Treatment of Influenza in Children (2024) [3]. Nevertheless, variation in drug selection and use in clinical practice continues to limit consistent treatment effectiveness and medication safety.

Translating the available evidence into knowledge that can be retrieved, understood, and applied in clinical practice remains challenging. Relevant information is distributed across clinical practice guidelines, expert consensus statements, randomized controlled trials, systematic reviews, and other clinical studies. These sources differ in terminology, syndrome classification, drug recommendations, dosing regimens, and safety information. Conventional retrieval from publications or guideline texts does not readily establish associations among these elements. Traditional Chinese medicine (TCM) also involves multiple interconnected dimensions, including clinical manifestations, syndrome differentiation, medicine selection, and conditions of use, which are difficult to represent fully in linear text. Large language models (LLMs) offer new opportunities for accessing medical information through natural language understanding and generation. However, they may generate information without clear evidentiary support, and the sources underlying their answers are often difficult to trace, limiting their trustworthy use in specialized medical settings [4,5].

Retrieval-augmented generation (RAG) combines external retrieval with language generation and offers a practical approach to grounding answers in available sources [6]. Medical benchmarks show that performance depends on retrieval configuration and task characteristics [7]. Graph-enhanced systems extend this approach by organizing biomedical entities and their relationships, as illustrated by iDISK2.0-RAG and MedGraphRAG [8,9]. A recent scoping review nevertheless found limited alignment between many medical RAG evaluations and actual clinical workflows [10].

### Study Objective

Knowledge graphs provide a means of integrating heterogeneous sources into a structured representation and visualizing complex associations [11,12]. They have been applied in clinical decision support for gastric cancer, diabetic kidney disease, and migraine. Their entity-relationship-attribute representation supports systematic knowledge management and reasoning [13-15]. In this study, we integrated evidence from clinical practice guidelines and other clinical sources to construct a structured, traceable knowledge graph of Chinese patent medicines for pediatric influenza. Evidence segmentation, ontology modeling, semiautomated knowledge extraction, terminology normalization, and knowledge integration were used to represent clinical manifestations, TCM syndromes, medicine recommendations, dosing, contraindications, adverse reactions, and evidence sources within a common semantic framework. We then developed a RAG question-answering system combining the Neo4j knowledge graph with an LLM and compared its answers with those of a standalone LLM using standardized questions. Figure 1 presents the overall study framework.

**Figure 1.**
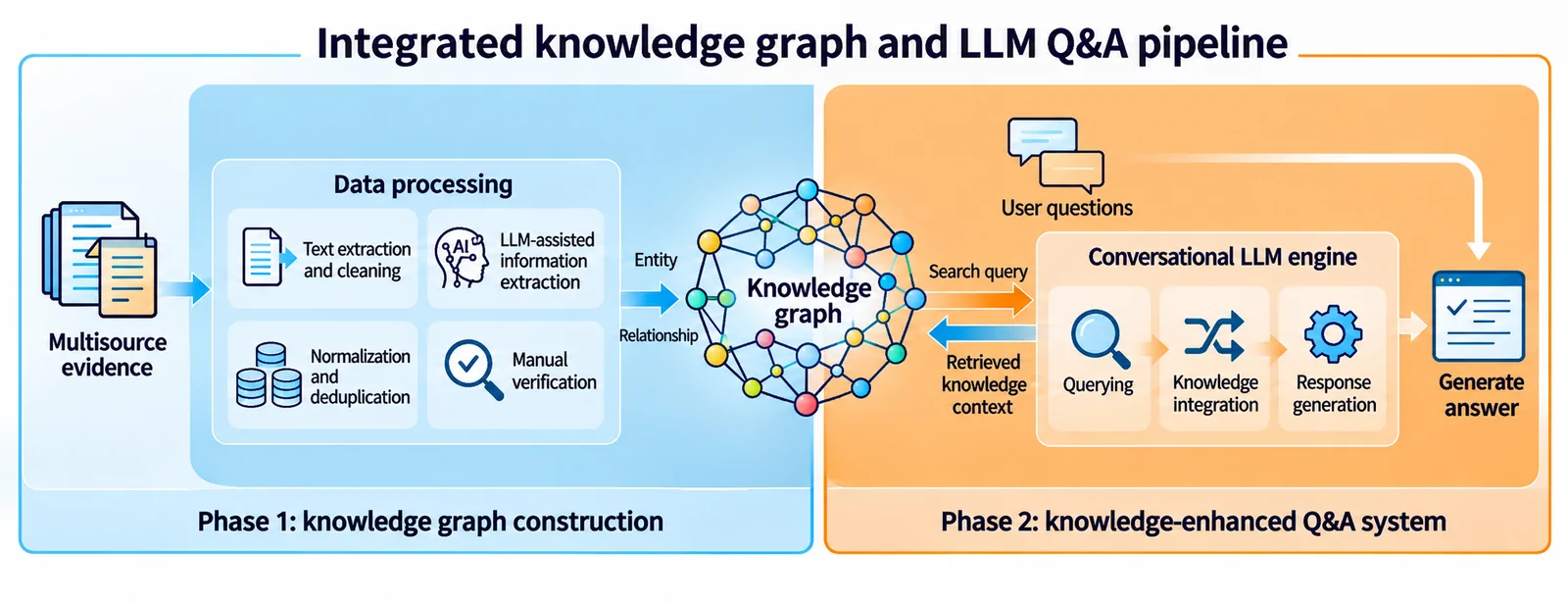
Architecture of the knowledge graph-enhanced question-answering system. The framework comprises knowledge graph construction, including LLM-assisted entity and relationship extraction and manual verification against established guidelines, and question-answering system development, in which the LLM combines user questions with relevant graph-retrieved knowledge to generate answers. LLM: large language model; Q&A: question answering.

## Methods

### Study Design

We developed a structured, traceable knowledge graph of Chinese patent medicines for pediatric influenza and a knowledge graph-enhanced LLM question-answering system. The knowledge engineering workflow comprised literature retrieval and evidence preprocessing, ontology development, knowledge extraction and integration, Neo4j graph construction, and development and evaluation of a graph-based RAG system. Reporting of the paired answer-quality evaluation was guided by the CHART (Chatbot Assessment Reporting Tool) guidelines (Checklist 1) [16].

### Literature Retrieval and Evidence Corpus Development

We conducted a systematic literature search to establish an evidence corpus for knowledge extraction and graph construction. PubMed, Embase, Web of Science Core Collection, the Cochrane Library, CNKI, Wanfang Data, and SinoMed were searched. We also searched relevant guideline repositories and professional society websites for eligible clinical practice guidelines and expert consensus statements. These supplementary searches yielded no additional unique eligible publications beyond those identified in the bibliographic databases. Search strategies combined controlled vocabulary and free-text terms related to pediatric influenza, TCM, and Chinese patent medicines and were adapted to each database. The search covered the period from September 2016 through September 2026, with the final search completed on September 4, 2026. Reference lists of eligible publications were also screened for additional evidence.

All records were imported into Zotero for management and deduplication, with manual checks based on titles, authors, and publication years. Two researchers independently screened titles and abstracts and assessed the full texts of potentially eligible publications. Disagreements were resolved through discussion or adjudication by a third researcher when necessary. Eligible studies involved children with influenza or influenza-like illness and provided clinical information on Chinese patent medicines that could populate the predefined knowledge schema. Relevant information included indications, syndromes or syndrome differentiation, clinical manifestations, treatment effects, dosing and administration, combination therapy, contraindications, adverse reactions, and other medication safety information. Eligible evidence types included clinical practice guidelines, expert consensus statements, randomized controlled trials, systematic reviews, meta-analyses, and observational studies. We excluded publications limited to populations other than children with influenza, studies without Chinese patent medicine recommendations or interventions, studies without extractable relevant information, animal experiments, and narrative reviews without extractable clinical evidence. Publications with unavailable full texts did not proceed to eligibility assessment.

The methodological quality of the 3 included clinical practice guidelines was assessed using the Appraisal of Guidelines for Research and Evaluation II (AGREE II) instrument [17]. Its framework was also used for descriptive assessments of 1 expert consensus statement and 2 prevention and treatment plans. Two researchers independently scored the 23 items across 6 domains and calculated standardized domain scores. The results were linked to the relevant publication nodes as source quality attributes in the knowledge graph to support documentation and traceability.

### Evidence Preprocessing

Full texts were converted to a consistent format and cleaned to produce a standardized corpus for knowledge extraction. Headers, footers, reference markers, and other layout elements unrelated to extraction were removed. Text displacement caused by multicolumn layouts, line breaks, or character encoding was corrected. Text was then segmented into evidence units, each representing a relatively self-contained clinical meaning. Each unit received a unique identifier, together with provenance information such as the source publication and section or location within that publication. We retained information relevant to the predefined scope: diseases, syndromes, symptoms and signs, Chinese patent medicines, indications, dosing and administration, treatment duration, combination therapy, contraindications, adverse reactions, and other safety information. Information outside the scope was excluded from subsequent extraction. Evidence units served as the smallest text inputs for knowledge extraction and remained linked to the original publications through unique source identifiers.

### Ontology Development

We used the 7-step ontology development method and Protégé 5.6.7 (Stanford University) to construct a domain ontology for Chinese patent medicines in pediatric influenza. The ontology served as the knowledge graph’s schema layer, defining core concepts, attributes, semantic relationships, and constraints on relationships between entity types. This framework standardized the representation of clinical terminology and semantic relationships, reduced terminological heterogeneity across sources, and provided semantic and structural constraints for entity recognition, relationship extraction, knowledge integration, and graph database storage [18].

### Knowledge Extraction and Integration

Knowledge extraction used a semiautomated approach combining LLM assistance with manual verification. Each preprocessed evidence unit was used as a text input, and the domain ontology supplied semantic constraints. Entities, relationships, and attributes were extracted from guidelines, expert consensus statements, randomized controlled trials, systematic reviews, meta-analyses, and observational studies according to the ontology’s definitions and connection rules. Publication identifiers, evidence unit identifiers, and original text were recorded concurrently to link the extracted knowledge to its underlying evidence [19,20].

The DeepSeek-Flash model was accessed through the official API. A consistent prompt template was developed from the predefined ontology and extraction rules, with standardized examples supplied through few-shot prompting. The model was instructed to extract only information explicitly stated in the input evidence unit, without adding, inferring, or generating information from external knowledge. No knowledge item was generated when the evidence was insufficient or its meaning could not be determined. Outputs followed a predefined JSON structure specifying fields for entities, relationships, attributes, and evidence sources. We used nonthinking mode, a temperature of 0.1, and a maximum output length of 2048 tokens to reduce randomness and improve consistency and reproducibility. Structured outputs underwent manual review of entity boundaries, entity types, semantic relationships, attribute values, and consistency with the original evidence before inclusion in the knowledge base.

Verified knowledge was normalized and integrated. Terminology normalization was guided by the Clinic Terminology of Traditional Chinese Medicine Diagnosis and Treatment—Part 1: Diseases (GB/T 16751.1‐2023), Part 2: Syndromes (GB/T 16751.2‐2021), and Part 3: Therapeutic Methods (GB/T 16751.3‐2023); the Pharmacopoeia of the People’s Republic of China; and the *ICD-11* (*International Classification of Diseases, 11th Revision*). Synonyms, near-synonyms, abbreviations, and alternative expressions were standardized, and entity alignment and deduplication reduced redundant knowledge [21-25]. Conflicting information from different sources was retained with its provenance rather than overwritten or deleted. Publication and evidence unit identifiers preserved source differences and the traceability of each knowledge statement.

### Knowledge Storage and Visualization

Neo4j (Neo4j Inc) was used to store and manage the integrated knowledge. Instances of diseases, syndromes, symptoms and signs, and Chinese patent medicines were represented as nodes. Semantic associations were represented as relationships, and entity characteristics and evidence information were stored as properties [26]. Publication identifiers, evidence unit identifiers, and other source information were retained during import to link the graph knowledge to the original evidence.

Graph creation, updating, and querying were implemented primarily in Cypher (Neo4j Inc). Reviewed and normalized entities, relationships, and attributes were imported in batches according to the predefined schema. Unique identifiers and entity matching rules supported node merging and relationship creation while limiting duplicate nodes and redundant relationships. Cypher pattern matching and path queries enabled retrieval across diseases, syndromes, clinical manifestations, medicines, and associated attributes, including multihop queries and tracing of evidence sources [27]. Neo4j visualization was used to display the resulting networks and evidence-based diagnostic and treatment knowledge paths.

### Development of the Graph-Based Question-Answering System

#### System Overview

We developed a RAG question-answering system to enable interactive retrieval and use of structured clinical knowledge. The system combined the natural language understanding and generation capabilities of an LLM with Neo4j’s structured retrieval capabilities. For questions about Chinese patent medicines in pediatric influenza, the system retrieved curated, traceable graph evidence rather than relying entirely on the model’s parametric knowledge. The Python implementation comprised question parsing, entity recognition and linking, structured graph retrieval, and evidence-constrained answer generation.

#### Intent Parsing and Entity Linking

The system used DeepSeek-Flash to parse each natural language question and identify information relevant to the ontology, including symptoms and signs, syndromes, patient characteristics such as age, and medicine-related, treatment-related, or safety-related query intents. To limit entity drift during generation, outputs were restricted to predefined entity types and query categories. Recognized entities were normalized and matched to standardized Neo4j nodes. Entities that could not be reliably matched were not forcibly linked.

#### Structured Graph Retrieval

After entity linking, the system selected a predefined Cypher template based on the recognized entities and query intent and passed the standardized entities as parameters to Neo4j. Templates covered medicine recommendations, indications, symptom-syndrome matching, dosing and administration, contraindications, adverse reactions, and evidence provenance. This design was intended to reduce syntax errors and retrieval path instability associated with unrestricted LLM-generated text-to-Cypher queries. Retrieval followed the node types and relationship paths defined in the ontology and returned a relevant subgraph containing entities, relationships, properties, and evidence sources. Age-related and dose-related information was further filtered using applicable graph properties and conditions. Publication and evidence unit identifiers were retained for provenance in the generated answers.

#### Evidence-Constrained Answer Generation

Retrieved entities, relationships, properties, and source information were converted into structured context and provided to the LLM together with the original question. Evidence-constrained prompts instructed the model to answer only from the retrieved graph content and not to supplement medicine names, doses, indications, or safety information with external knowledge. When graph evidence was insufficient, the system was required to state that limitation rather than infer an answer. Responses followed a predefined format and, as appropriate to the query, presented medicine names, indications or syndromes, dosing and administration, contraindications, adverse reactions, and evidence sources, retaining links to the original evidence.

Both query parsing and answer generation used DeepSeek-Flash with a temperature of 0.1 and a maximum answer length of 1024 tokens. Retrieved structured knowledge was supplied as context. A Streamlit web interface supported natural language questions, graph retrieval, and display of evidence-constrained answers. Figure 2 summarizes the workflow.

**Figure 2.**
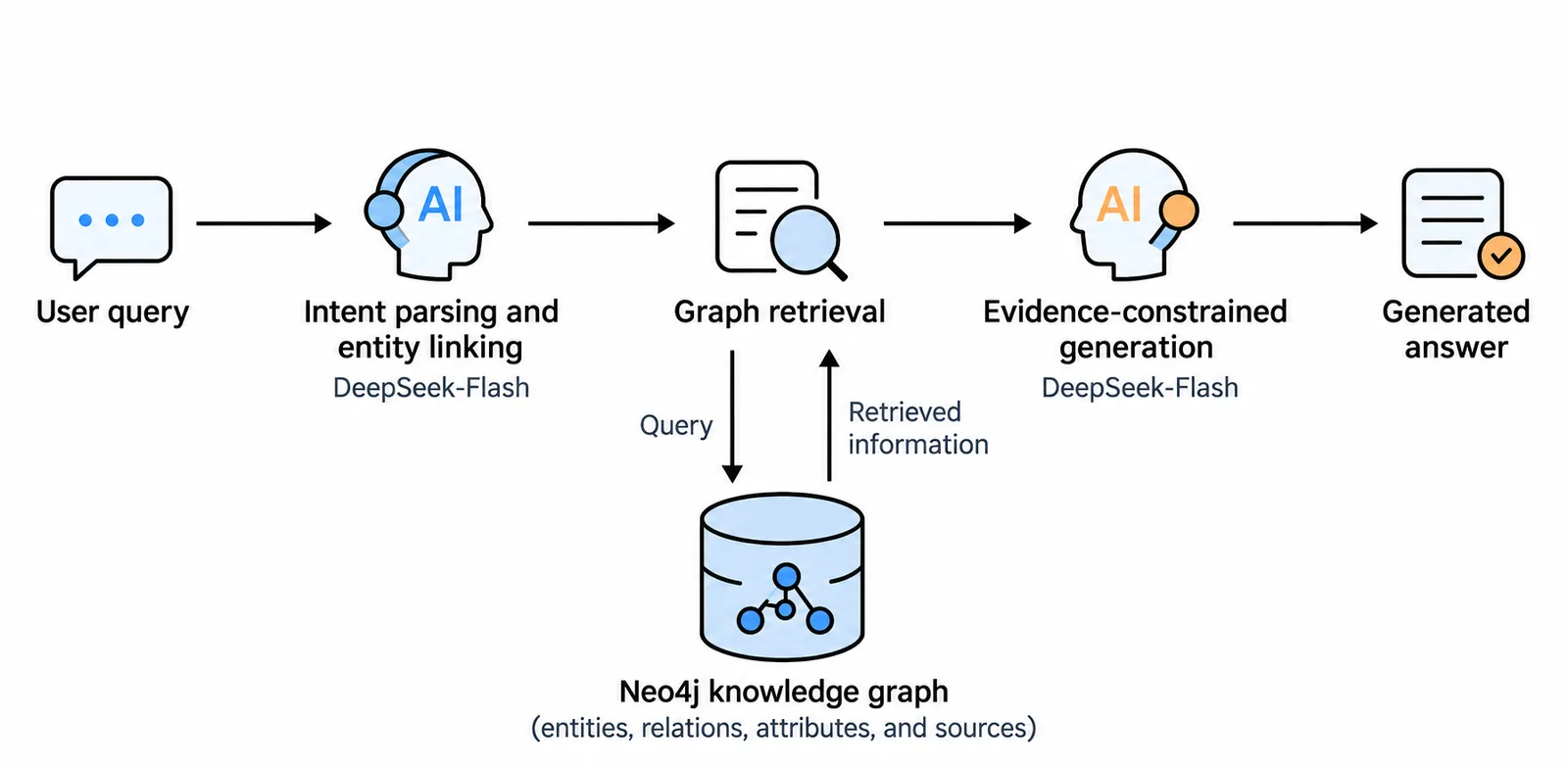
Workflow of the knowledge graph–based RAG question-answering system. Queries undergo intent parsing, entity recognition, and entity linking. Predefined Cypher templates (Neo4j, Inc) retrieve a relevant evidence subgraph from Neo4j, including entities, relationships, properties, and sources. The retrieved information and original question are supplied to DeepSeek-Flash for evidence-constrained answer generation, retaining provenance links. RAG: retrieval-augmented generation.

### Evaluation of Answer Quality

We developed a standardized evaluation set from the clinical evidence incorporated into the knowledge graph. The research team designed questions using guidelines, expert consensus statements, and structured evidence. The 80 questions comprised medicine recommendations (n=24), evidence reasoning (n=20), evidence provenance (n=20), and safety (n=16). They addressed syndrome differentiation, therapeutic principles, medicine recommendations, dosing and administration, evidentiary support and provenance, contraindications, and adverse reactions. Reference answers were based on the original publications and evidence units specified the core assessment criteria and acceptable answers for each question.

Each question was submitted to a standalone LLM and a knowledge graph–enhanced LLM (KG-LLM). Each model generated 1 answer per question, yielding 160 answers and 80 pairs. Both configurations used DeepSeek-Flash with a temperature of 0.1 and a maximum answer length of 1024 tokens. The standalone LLM did not retrieve graph knowledge or receive graph-derived evidence context. KG-LLM followed the predefined Neo4j retrieval workflow and generated answers from the retrieved knowledge and source evidence. Before assessment, answers were randomly ordered and assigned anonymous codes. Three experts with clinical experience in TCM pediatrics independently rated the answers while blinded to their model of origin.

Answer quality was assessed using core task accuracy, completeness, and unsupported factual content. Accuracy measured the correctness of core medical facts and task conclusions. Completeness measured coverage of the key information requested by the question. Both were scored from 0 to 5, with higher scores indicating better performance. Unsupported factual content was defined as the presence of at least 1 factual statement without sufficient evidentiary support and was classified as present, not identified, or indeterminate.

Each answer was independently assessed by all 3 experts. Final accuracy and completeness scores were the arithmetic means of the 3 ratings. For unsupported factual content, a majority rule specified before unblinding was applied: a determinate judgment shared by at least 2 experts was used as the final classification; otherwise, the answer remained indeterminate. All individual ratings were retained for interrater reliability analysis.

### Exploratory End-User Usability Evaluation

Five physicians from different specialties participated in a small exploratory evaluation of usability and user experience. Following standardized instructions, participants used the web interface for approximately 10 to 15 minutes, independently posed questions about Chinese patent medicines for pediatric influenza, reviewed answers and evidence sources, and attempted to locate the corresponding publications and evidence passages in an accompanying repository of original PDFs. Overall usability was assessed with the 10-item System Usability Scale (SUS) [28]. Items used a 5-point response scale and were converted to a score from 0 to 100 according to standard scoring rules, with higher scores indicating greater perceived usability. Six study-specific 5-point Likert items assessed ease of use, perceived usefulness, ease of locating evidence, ease of verifying evidence, comprehensibility, and intention to use. Participants also selected a self-reported evidence-location outcome and could provide optional suggestions. This evaluation assessed subjective experience and self-reported evidence location; it did not objectively validate provenance accuracy or clinical outcomes.

### Statistical Analysis

Descriptive and paired inferential analyses compared the standalone LLM and KG-LLM. Accuracy and completeness were summarized as means and SDs. Answers to the same question formed the unit of pairing. Between-model differences were assessed using the Wilcoxon signed-rank test, with the matched-pairs rank-biserial correlation calculated as an effect size. Unsupported factual content was compared using the exact McNemar test, restricted to pairs in which both answers received determinate classifications.

Intraclass correlation coefficients (ICCs) assessed agreement among the 3 experts for accuracy and completeness [29], and Fleiss κ assessed agreement for unsupported factual content [30]. Bootstrap methods were used to estimate 95% CIs for paired differences. Sensitivity analyses examined the influence of indeterminate records. The Holm method adjusted the 3 primary comparisons for multiple testing, with adjusted *P*<.05 considered statistically significant.

Usability outcomes were summarized descriptively. SUS scores were reported using the mean and SD, median and IQR (25th-75th percentiles), and range. Quartiles were calculated using linear interpolation. Each study-specific Likert item was reported separately using the mean and SD; no composite score was calculated. Self-reported evidence-location outcomes were summarized as counts and percentages, and open-ended feedback was summarized thematically. No inferential testing was conducted for the usability evaluation because of its small sample. Analyses of model answer quality were performed in Python (version 3.13).

### Knowledge Graph Updating

A version-controlled updating mechanism was designed to support long-term maintenance and alignment with emerging clinical evidence. Newly published or revised guidelines, expert consensus statements, and other relevant high-quality evidence would undergo the same source registration, evidence segmentation, and ontology-constrained extraction processes used in initial construction. Candidate knowledge would be compared with the current knowledge base and classified as new, unchanged, modified, or conflicting before manual verification and incorporation. Each knowledge item would retain its source publication, evidence unit identifier, validity period, update status, and replacement relationships. Superseded knowledge would be marked as inactive or replaced rather than deleted, preserving the historical record. Each update would receive a unique version number and a record of the evidence cutoff date and principal changes, supporting current knowledge retrieval, historical tracing, and rollback when needed.

### Ethical Considerations

Evidence extraction, knowledge graph construction, and system development used published and lawfully accessed clinical practice guidelines, expert consensus statements, and clinical studies. These activities did not involve recruitment of children, clinical intervention, collection of human biological samples, or collection of identifiable patient information. The authors’ rationale for not seeking ethics review of the literature-based component was Article 32 of China’s Measures for Ethical Review of Life Science and Medical Research Involving Human Subjects, which provides for exemption from ethics review for public-data research meeting specified conditions [31].

Three experts in TCM pediatrics independently evaluated model answers under blinded conditions, and 5 physicians participated in the exploratory usability evaluation. No formal ethics approval or exemption determination was obtained for these evaluations. Physicians participating in the usability evaluation were informed in advance that their evaluation data would be used in a research publication and participated voluntarily. The evaluations concerned model answer quality and system use and did not involve treatment decisions for actual patients or clinical outcomes.

## Results

### Literature Retrieval and Evidence Corpus Development

Database searches identified 1314 records. After deduplication, 727 records remained; following title and abstract screening and full-text retrieval, 212 publications underwent eligibility assessment, and 142 were included. The corpus comprised 3 clinical practice guidelines, 1 expert consensus statement, 2 prevention and treatment plans, 113 randomized controlled trials, 7 systematic reviews or meta-analyses, and 16 other studies. Searches of guideline repositories, professional society websites, and reference lists yielded no additional unique eligible publications. Figure 3 presents the selection process.

**Figure 3.**
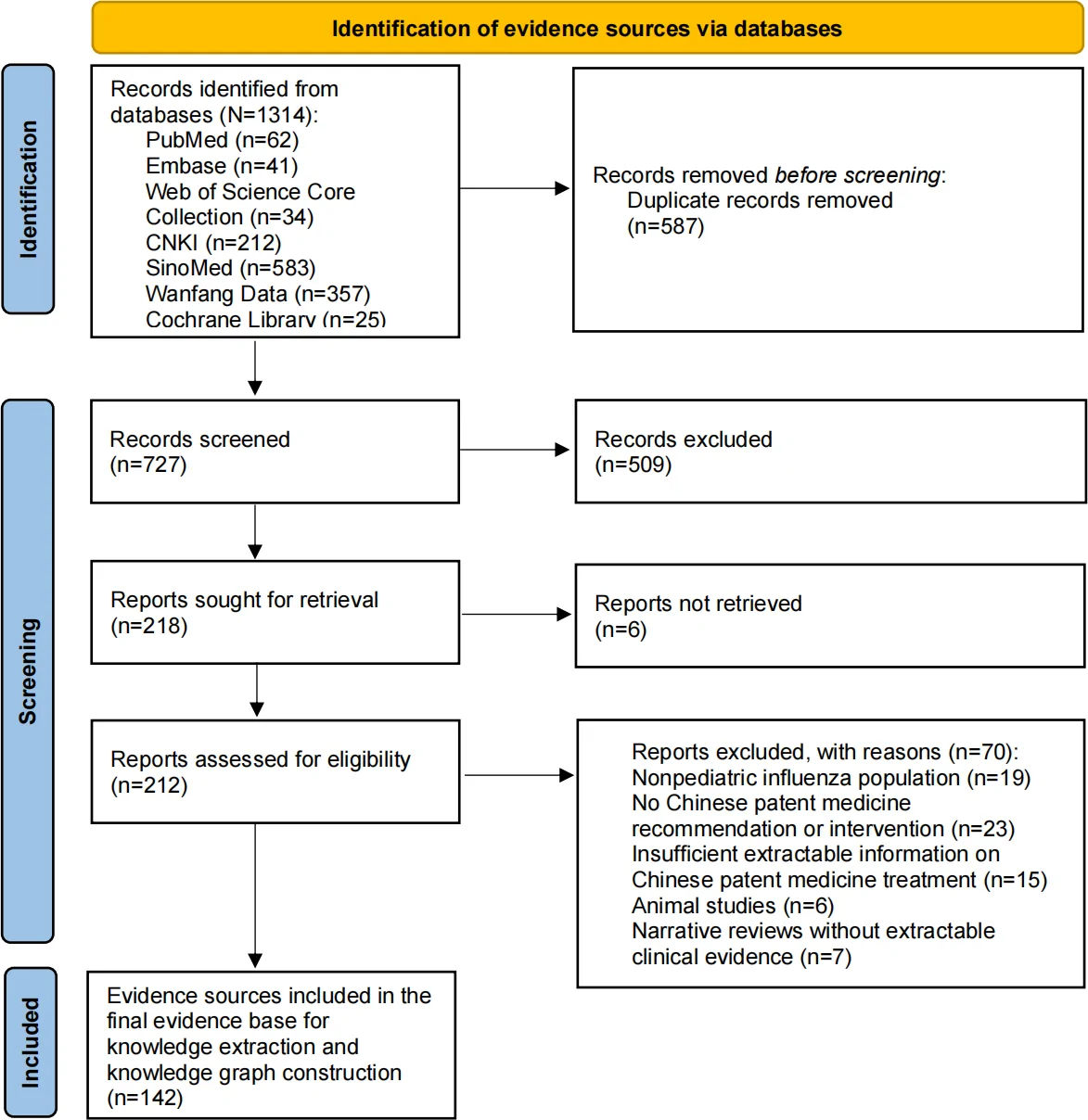
Selection of publications for the evidence corpus (142 included).

AGREE II assessments of the 3 guidelines and descriptive assessments of the consensus statement and 2 prevention and treatment plans indicated relatively higher scores for scope and purpose and clarity of presentation, and lower scores for rigor of development and applicability. Assessment results were incorporated as source quality attributes in the graph.

Format conversion, text cleaning, and structured segmentation yielded 1294 evidence units. Unique identifiers linked each unit to its source publication and original text location, supporting extraction, integration, and provenance.

### Ontology and Semantic Structure

The schema layer organized core concepts, properties, and semantic relationships within the predefined evidence scope. Knowledge dimensions included disease and diagnosis, clinical manifestations, TCM syndrome differentiation, Chinese patent medicine treatment, medication regimens, safety, and evidence sources. Disease-related knowledge included influenza diagnosis, severity, and differential diagnosis. Clinical manifestations included symptoms, signs, and tongue and pulse findings. Syndrome differentiation linked TCM syndromes to their clinical manifestations. Medicine-related knowledge included applicable syndromes or symptoms, ingredients, dosing and administration, treatment duration, and combination therapy. Safety knowledge covered contraindications, adverse reactions, and precautions. Evidence-related concepts captured recommendation strength, evidence level, source publications, and evidence units.

Permitted semantic relationships and connection constraints were defined for entity types. For example, clinical manifestations were linked to TCM syndromes, syndromes to appropriate Chinese patent medicines, and medicines to dosing, safety information, and supporting evidence. Distinguishing classes, instances, attributes, and relationships allowed individual medicines, syndromes, symptoms, and evidence records to be represented consistently. The schema constrained LLM-assisted extraction and supported normalization, entity alignment, Neo4j instantiation, and provenance. Figure 4 illustrates the conceptual structure of the knowledge graph schema.

**Figure 4.**
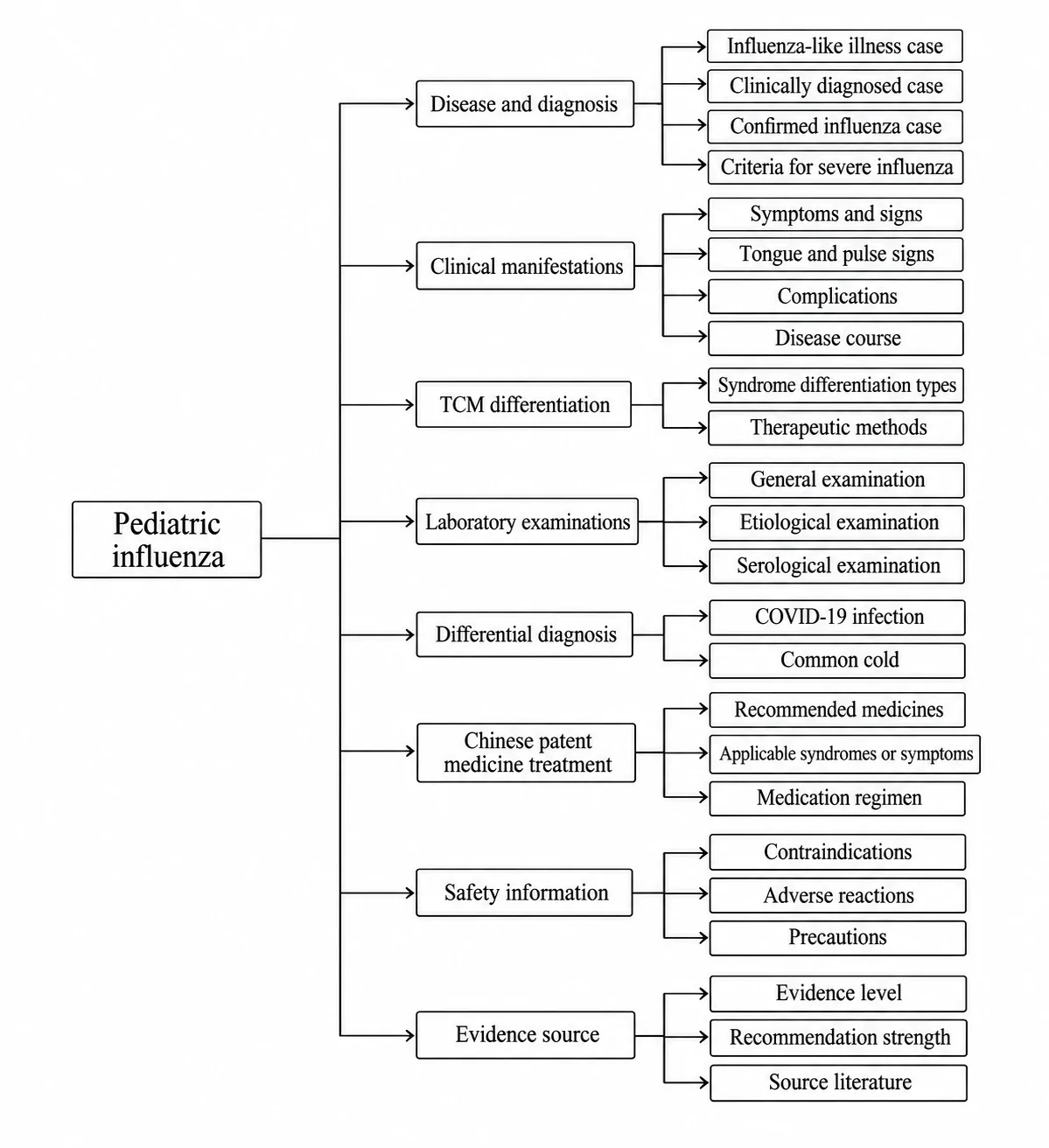
Conceptual structure of the schema layer for the pediatric influenza knowledge graph. Core categories and their hierarchical relationships cover disease and diagnosis, clinical manifestations, TCM differentiation, laboratory examinations, differential diagnosis, Chinese patent medicine treatment, safety information, and evidence sources. TCM: traditional Chinese medicine.

### Knowledge Extraction, Normalization, and Provenance

LLM-assisted extraction and manual verification were applied to all 1294 standardized evidence units. After verification, terminology normalization, entity alignment, and deduplication, 3211 standardized entities and 19,671 valid semantic relationships were retained.

Synonyms, abbreviations, and alternative names for diseases, syndromes, symptoms, and medicines were mapped to standardized concepts. Original expressions were retained to preserve correspondence with the source text. Differences in recommendations, doses, treatment duration, or safety information across publications were preserved as separate knowledge statements with their respective sources.

Provenance was recorded during extraction. Publication and evidence unit identifiers linked each knowledge record to the original evidence, allowing users to trace graph knowledge to individual publications and specific passages through a chain of structured knowledge, evidence units, and source publications.

### Knowledge Graph Construction

Importing the normalized and integrated data into Neo4j produced a graph with 3211 nodes and 19,671 relationships across 23 entity types and 38 relationship types. The graph organized dispersed clinical information into a queryable network connecting clinical manifestations, TCM syndromes, recommended Chinese patent medicines, dosing, safety information, and evidence sources. Cypher path queries supported multihop retrieval and identification of the publications and evidence units underlying specific knowledge. Figures 5-7 show the overall graph and representative subgraphs.

**Figure 5.**
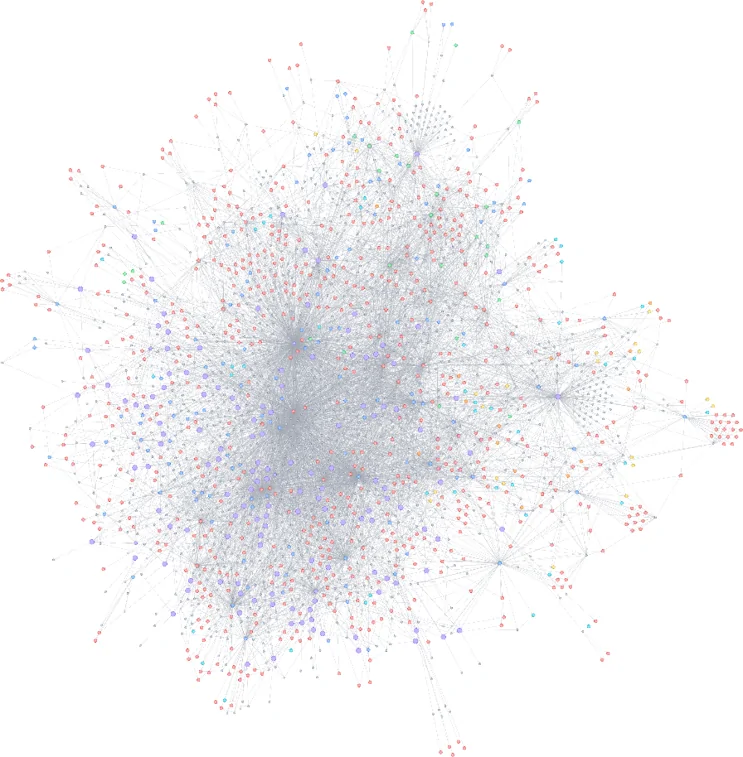
Overall structure of the pediatric influenza knowledge graph in Neo4j. The network contains 3211 nodes and 19,671 relationships. Nodes represent different entity categories, including diseases, clinical manifestations, TCM syndromes, Chinese patent medicines, medication information, safety knowledge, and evidence sources. TCM: traditional Chinese medicine.

**Figure 6.**
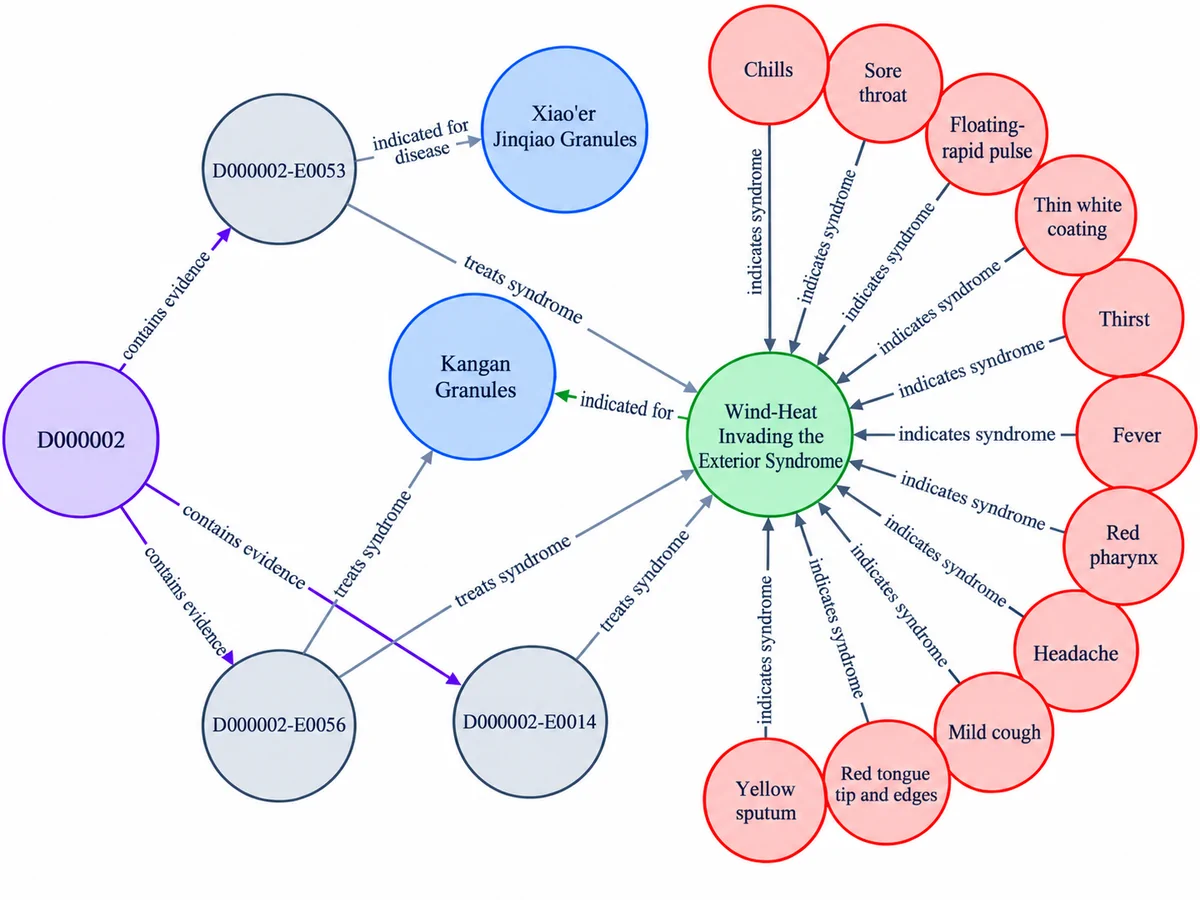
Representative subgraph linking a TCM syndrome, clinical manifestations, and Chinese patent medicines. The central green node represents the wind-heat invading the exterior syndrome; pink nodes represent clinical manifestations, blue nodes represent medicines, gray nodes represent evidence units, and the purple node represents a source publication. The medicines shown are Xiao’er Jinqiao Granules and Kanggan Granules. TCM: traditional Chinese medicine.

**Figure 7.**
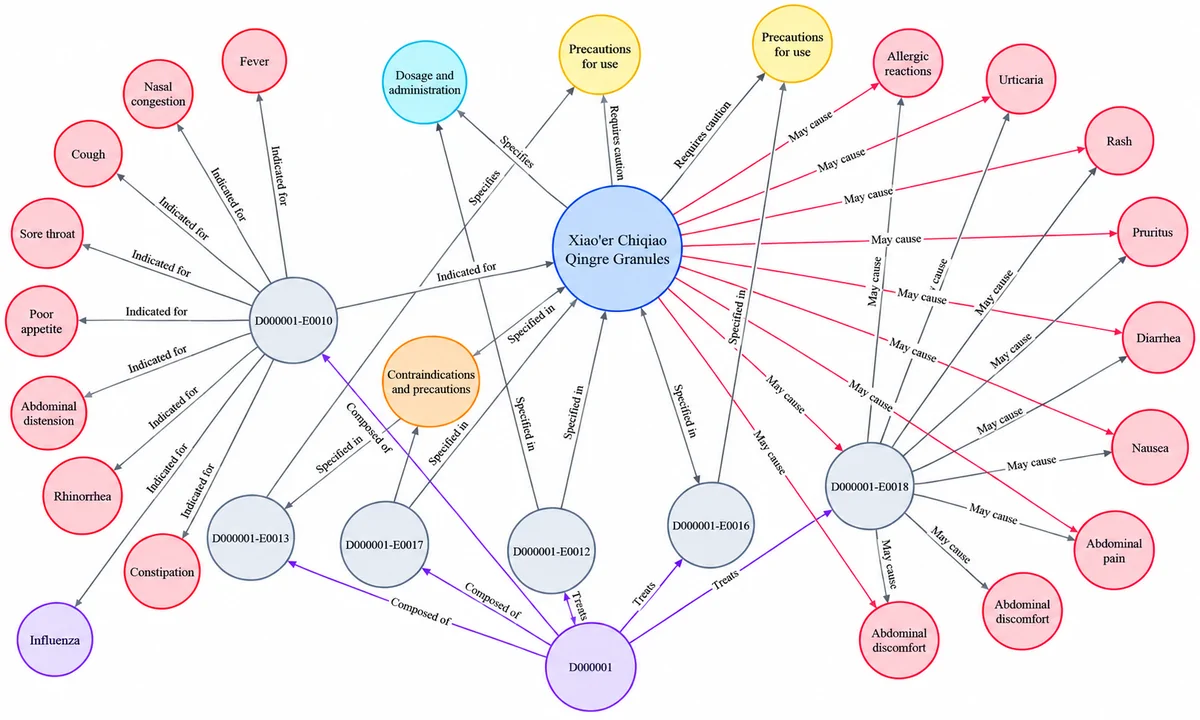
Representative subgraph of medicine use and evidence provenance. The subgraph connects a Chinese patent medicine to clinical and medication-related information and source evidence. Evidence unit and publication nodes link structured knowledge to the underlying evidence.

### Implementation of the Question-Answering System

The graph-enhanced RAG system supported queries about medicine recommendations, symptom-syndrome matching, dosing and administration, combination therapy, contraindications, adverse reactions, and evidence sources. Natural language questions were mapped to standardized graph entities, and retrieved knowledge was returned with its source evidence. The system indicated insufficient evidence when the graph could not adequately support an answer. Figure 8 shows the interface and an example interaction.

**Figure 8.**
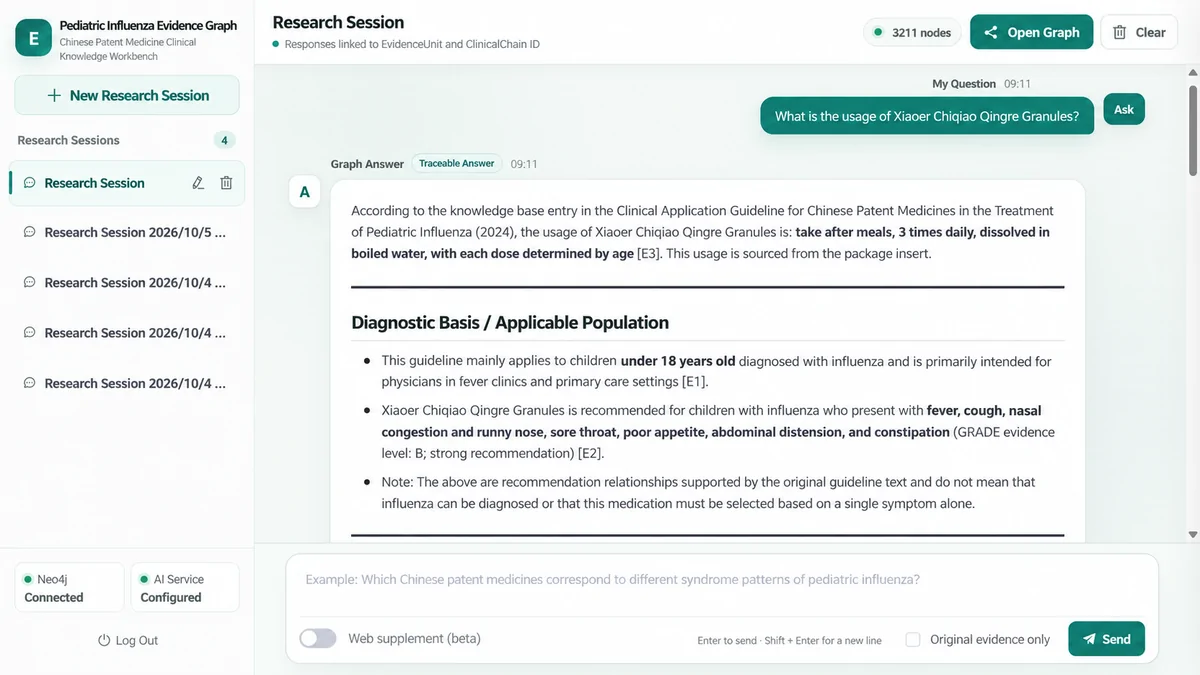
User interface of the knowledge graph–enhanced question-answering system. The example interaction concerns the use of Xiao’er Chiqiao Qingre Granules. The interface displays an answer and source identifiers, illustrating how a question is connected to retrieved evidence. The screenshot reproduces an example system output.

### Evaluation of Answer Quality

The 80 standardized questions yielded 80 answers from each model, forming 80 pairs. KG-LLM achieved higher mean accuracy and completeness scores than the standalone LLM.

Mean accuracy was 3.05 (SD 1.88) for KG-LLM and 2.02 (SD 1.54) for the standalone LLM, with a mean paired difference of 1.02 (95% CI 0.59‐1.45). Mean completeness was 3.11 (SD 1.84) and 2.42 (SD 1.62), respectively, with a mean paired difference of 0.70 (95% CI 0.26‐1.12). Wilcoxon signed-rank tests indicated statistically significant differences after Holm adjustment (*P*<.001 for accuracy and *P*=.002 for completeness).

Two pairs had indeterminate assessments of unsupported factual content, leaving 78 complete pairs for analysis. Unsupported factual content was identified in 11 standalone LLM answers (n=11, 14.1%) and no KG-LLM answers (n=0, 0%). The difference in proportions for KG-LLM minus the standalone LLM was −14.1 percentage points (95% CI −21.8 to −6.4). The exact McNemar test indicated a significant difference after Holm adjustment (*P*=.002). Extreme-case sensitivity analyses for the 2 indeterminate answers also retained statistical significance.

Interrater agreement was high. The average-measures ICCs for the 3 experts were 0.984 for accuracy and 0.975 for completeness. Fleiss κ for unsupported factual content was 0.759 among 154 answers with determinate binary assessments. Table 1 summarizes the primary comparisons.

**Table 1.** Answer quality of the standalone LLM^a^ and KG-LLM^b^ on standardized questions.

Outcome	Standalone LLM	KG-LLM	Adjusted *P* value^c^
Core task accuracy, mean (SD)^d^	2.02 (1.54)	3.05 (1.88)	<.001
Completeness, mean (SD)^d^	2.42 (1.62)	3.11 (1.84)	.002
Unsupported factual content, n/N (%)^e^	11/78 (14.1)	0/78 (0)	.002

a LLM: large language model.

b KG-LLM: knowledge graph–enhanced large language model.

c All *P* values were adjusted using the Holm method across the 3 primary comparisons.

d Scores range from 0 to 5. Accuracy and completeness each included 80 pairs and were compared using the Wilcoxon signed-rank test.

e Presence of at least 1 unsupported factual statement. The exact McNemar test included 78 pairs with determinate assessments for both models.

### Exploratory End-User Usability Evaluation

All 5 physicians completed the usability evaluation: 2 pediatricians practicing Western medicine, 1 TCM pediatrician, 1 physician from another TCM specialty, and 1 general practitioner. The mean SUS score was 96.50 (SD 2.85), the median was 97.50 (IQR 95.00-97.50), and scores ranged from 92.50 to 100.00.

Mean scores for ease of use, perceived usefulness, and ease of locating evidence were each 5.00 (SD 0.00). The mean score was 4.60 (SD 0.55) for ease of verifying evidence and 4.80 (SD 0.45) for both comprehensibility and intention to use. Of the 5 participants, 4 (80%) reported locating both the original publication and the corresponding evidence passage, whereas 1 participant (20%) located the publication but not the specific passage. Open-ended feedback suggested expanding treatment information and improving the display of original evidence excerpts. Table 2 presents the results.

**Table 2.** Exploratory end-user usability outcomes among 5 physicians.

Outcome	Result
SUS^b^ score, mean (SD)	96.50 (2.85)
SUS score, median (IQR^c^)	97.50 (95.00‐97.50)
Study-specific Likert items, mean (SD)^d^	
Ease of use	5.00 (0.00)
Perceived usefulness	5.00 (0.00)
Ease of locating evidence	5.00 (0.00)
Ease of verifying evidence	4.60 (0.55)
Comprehensibility	4.80 (0.45)
Intention to use	4.80 (0.45)
Self-reported evidence location, n/N (%)^e^	
Located the publication and corresponding evidence passage	4/5 (80)
Located the publication but not the specific evidence passage	1/5 (20)

b SUS: System Usability Scale. Scores range from 0 to 100.

c IQR reported as the 25th-75th percentiles.

d Each item was rated on a 5-point scale; higher scores indicate more favorable ratings. Items were analyzed separately.

e Evidence-location outcomes were self-reported and were not independently verified.

## Discussion

### Principal Findings

This study developed a traceable knowledge graph and a graph-enhanced question-answering system for Chinese patent medicines in pediatric influenza. The graph integrates symptoms, TCM syndromes, medicines, dosing and administration, treatment duration, contraindications, adverse reactions, and provenance. Knowledge is organized along clinical manifestation-syndrome-treatment pathways, reflecting the logic of syndrome differentiation and treatment. Medicine recommendations are linked to applicable populations, dosing regimens, treatment duration, evidence levels, and recommendation strength. Explicit links to safety information and source evidence provide a foundation for structured clinical knowledge retrieval and decision support.

### Comparison With Prior Work and Methodological Contributions

Existing disease-specific knowledge graphs have supported gastric cancer treatment recommendations, diabetic kidney disease knowledge discovery, and migraine knowledge representation [13-15]. More closely related TCM question-answering studies include the Duan et al [20] system based on historical case records and the Chen et al [32] Jin San Zhen therapy system. These precedents show that combining graph retrieval with LLM generation is an established research direction. The present study applies this approach to a defined pediatric influenza medicine domain, emphasizing links among clinical manifestations, syndromes, dosing, safety information, and supporting evidence. Outside TCM, DR.KNOWS used relevant graph paths to support diagnosis generation from electronic health records [33]. Differences in clinical domain and evaluation task should be considered when interpreting these related systems.

Evidence provenance is also addressed in prior systems, including iDISK2.0-RAG, MedGraphRAG, and the Jin San Zhen question-answering system [8,9,32]. In our implementation, publication identifiers, evidence unit identifiers, and source text were recorded during extraction and preserved throughout integration and storage, allowing graph knowledge to be traced to specific passages. Conflicting statements were retained with their respective sources, preserving heterogeneity across the underlying evidence.

The system also used predefined Cypher templates selected by query intent rather than unrestricted LLM-generated queries. The LLM was used chiefly for natural language understanding, entity recognition, and answer formulation, while retrieval of medical knowledge was constrained by predefined graph paths and query rules. This design was intended to reduce retrieval drift, syntax errors, and irreproducibility. Retrieved knowledge and provenance were supplied as context, and evidence-constrained prompts restricted answers to retrieved content. When the graph provided insufficient support, the system reported insufficient evidence instead of supplementing the answer with parametric knowledge.

### Effects of Graph Enhancement on Answer Quality

Across 80 paired questions, KG-LLM achieved significantly higher accuracy and completeness than the standalone LLM. These findings suggest that structured retrieval and evidence constraints can improve performance on a defined medical question-answering task. Ontology-constrained relationships among clinical manifestations, syndromes, medicine recommendations, dosing, and safety information, together with source links and predefined queries, provided question-relevant context and reduced reliance on parametric knowledge.

The direction of our findings is consistent with source-grounded clinical question answering in Almanac and graph-based question answering in iDISK2.0-RAG [4,8]. However, different corpora, question formats, model configurations, and outcome measures preclude direct comparisons of performance. The MIRAGE benchmark also shows that retrieval benefits are task- and configuration-dependent [7].

KG-LLM also generated less unsupported factual content. Among 78 evaluable pairs, 11 standalone LLM answers contained at least 1 unsupported factual statement, whereas none were identified in KG-LLM answers. This finding may reflect the evidence-constrained generation strategy: provenance links supplied source-grounded context, and insufficient-evidence responses limited unsupported supplementation. However, these results do not establish that knowledge graphs eliminate LLM hallucinations or demonstrate improved safety in clinical practice.

Evidence support, therefore, warrants separate assessment from overall task accuracy. Retrieve-Then-Verify identified unsupported claims despite relatively strong task-level performance, although it evaluated already generated outputs rather than a generation-time RAG intervention [5]. In a drug safety auditing study, explicitly enforced evidence boundaries reduced unsupported reassurance, with the boundary determined by the supplied evidence and prompting policy [34]. These studies reinforce the need to retain an insufficient-evidence response and evaluate source support directly.

The findings support using knowledge graphs as an external retrieval resource and knowledge constraint for medical question answering within a predefined evidence scope. Nevertheless, accuracy and completeness remained improvable. We did not separately evaluate the contributions of graph retrieval, evidence-constrained prompting, or other system components. The results therefore characterize the combined framework’s technical performance on predefined tasks; its value for complex questions and actual clinical care requires further investigation.

### Usability and Evidence-Provenance Experience

The exploratory evaluation among 5 physicians suggested high perceived usability during brief use. Participants rated ease of use, perceived usefulness, and ease of locating evidence favorably. Although all 5 reported locating source publications, 1 could not locate the specific evidence passage, and ease of evidence verification received a comparatively lower score. Providing source information therefore does not necessarily make verification straightforward. More precise location information and improved presentation of source excerpts may improve the experience. These findings provide preliminary evidence of usability but do not establish workflow efficiency or effects on clinical decisions.

### Limitations

The study has several limitations. First, although ontology constraints and manual verification supported consistent knowledge extraction, the representation of complex clinical meaning may still have been influenced by model performance and human judgment. Errors in graph contents or the selection of irrelevant paths may propagate into answers, as illustrated by the error analysis in DR.KNOWS [33]. Second, evaluation questions and reference answers were developed primarily from the same evidence base used to construct the graph. This was an internal evaluation within a defined evidence scope and may not capture the complexity of real clinical questions. Third, the small usability sample may be subject to selection and subjective assessment biases. The evaluations were not conducted within actual clinical workflows. Generalizability, long-term usability, and clinical safety remain to be established.

### Future Directions

Future work should validate the system using independent test sets, multiple institutions, and different user groups. Such studies could assess usability, evidence retrieval efficiency, user acceptance, and stability across settings and explore integration with electronic health records or clinical information systems. Applications involving actual treatment decisions or patient outcomes require prospective, multicenter evaluation of effectiveness and safety. Future studies conducted in real clinical settings should use an appropriate reporting framework, such as DECIDE-AI (Developmental and Exploratory Clinical Investigations of Decision Support Systems Driven by Artificial Intelligence) for early-stage live clinical evaluation [35]. The version-control and provenance mechanisms could support regular incorporation of new or revised guidelines, consensus statements, and other high-quality evidence, with conflict checking to maintain currency. Further work combining natural language processing and graph reasoning may improve representation of complex syndromes, changing clinical conditions, and individualized information while retaining source traceability and evidence constraints.

### Conclusions

We integrated clinical evidence on Chinese patent medicines for pediatric influenza through evidence segmentation, ontology development, and knowledge extraction and integration to construct a structured, traceable knowledge graph and a graph-enhanced LLM question-answering system. The system supported interactive retrieval and provenance through structured graph queries and evidence-constrained generation. On standardized questions, KG-LLM achieved higher accuracy and completeness and less unsupported factual content than a standalone LLM. A small exploratory evaluation also indicated high perceived usability. This framework offers a feasible approach to organizing clinical knowledge, tracing evidence, and supporting source-grounded question answering, while its generalizability, practical effectiveness, and safety in clinical care require further validation.

## Supplementary material

10.2196/101648 Checklist 1 CHART checklist.

## Abbreviations

AGREE II: Appraisal of Guidelines for Research and Evaluation II
CHART: Chatbot Assessment Reporting Tool
DECIDE-AI: Developmental and Exploratory Clinical Investigations of Decision Support Systems Driven by Artificial Intelligence
ICC: intraclass correlation coefficient
*ICD-11*: *International Classification of Diseases, 11th Revision*
KG-LLM: knowledge graph–enhanced large language model
LLM: large language model
RAG: retrieval-augmented generation
SUS: System Usability Scale
TCM: traditional Chinese medicine

## References

[1] National Health Commission of the People’s Republic of China, National Administration of Traditional Chinese Medicine (2025). Influenza diagnosis and treatment protocol (2025 Edition) [Article in Chinese]. Chin J Infect Control.

[2] Yang Z, Yan Y, Wu X (2021). Meta-analysis of five kinds of Qingrejiedu Chinese medicine combined with oseltamivir on treatment of influenza in children [Article in Chinese]. Drug Eval Res.

[3] Shi NN, Xiong L, Ma R (2025). Clinical application guidelines for traditional Chinese medicine in the treatment of childhood influenza (2024) [Article in Chinese]. J Tradit Chin Med.

[4] Zakka C, Shad R, Chaurasia A (2024). Almanac - retrieval-augmented language models for clinical medicine. NEJM AI.

[5] Liang Z, Sheffield C, Butera G, Antani S (2026). Retrieve-then-verify for evaluating evidence support and hallucination in large language model-generated medical information: empirical study. JMIR AI.

[6] Lewis P, Perez E, Piktus A, Larochelle H, Ranzato M, Hadsell R, Balcan MF, Lin H (2020). NIPS ’20: Proceedings of the 34th International Conference on Neural Information Processing Systems.

[7] Xiong G, Jin Q, Lu Z, Zhang A (2024). Benchmarking retrieval-augmented generation for medicine. Findings Assoc Comput Linguist ACL.

[8] Hou Y, Bishop JR, Liu H, Zhang R (2025). Improving dietary supplement information retrieval: development of a retrieval-augmented generation system with large language models. J Med Internet Res.

[9] Wu J, Zhu J, Qi Y (2025). Medical graph RAG: evidence-based medical large language model via graph retrieval-augmented generation. Proc Annu Meet Assoc Comput Linguist.

[10] Miao Y, Zhao Y, Luo Y, Wang H, Wu Y (2025). Improving large language model applications in the medical and nursing domains with retrieval-augmented generation: scoping review. J Med Internet Res.

[11] Chandak P, Huang K, Zitnik M (2023). Building a knowledge graph to enable precision medicine. Sci Data.

[12] Hogan A, Blomqvist E, Cochez M (2022). Knowledge graphs. ACM Comput Surv.

[13] Li S, Li Z, Xue K (2024). GC-CDSS: personalized gastric cancer treatment recommendations system based on knowledge graph. Int J Med Inform.

[14] Zhao X, Wang Y, Li P (2023). The construction of a TCM knowledge graph and application of potential knowledge discovery in diabetic kidney disease by integrating diagnosis and treatment guidelines and real-world clinical data. Front Pharmacol.

[15] Jiang Y, Qiu S, Guo Z (2023). Construction of migraine knowledge graph based on diagnosis and treatment guidelines of integrative medicine. Chinese Med Nat Prod.

[16] CHART Collaborative (2025). Reporting guideline for chatbot health advice studies: the Chatbot Assessment Reporting Tool (CHART) statement. BMJ Med.

[17] Brouwers MC, Kho ME, Browman GP (2010). AGREE II: advancing guideline development, reporting and evaluation in health care. CMAJ.

[18] Noy NF, McGuinness DL (2001). Ontology development 101: a guide to creating your first ontology. https://protege.stanford.edu/publications/ontology_development/ontology101.pdf.

[19] Mo B, Yu K, Kazdan J (2025). KGGen: extracting knowledge graphs from plain text with language models. Adv Neural Inf Process Syst.

[20] Duan Y, Zhou Q, Li Y (2025). Research on a traditional Chinese medicine case-based question-answering system integrating large language models and knowledge graphs. Front Med (Lausanne).

[21] State Administration for Market Regulation, Standardization Administration of China (2023). Clinic terminology of traditional Chinese medical diagnosis and treatment—part 1: diseases (GB/t 16751.1-2023) [Report in Chinese]. https://openstd.samr.gov.cn/bzgk/std/newGbInfo?hcno=7377FB06FF43DC321BB6A4BF7DAA7F39.

[22] State Administration for Market Regulation, Standardization Administration of China (2021). Clinic terminology of traditional Chinese medical diagnosis and treatment—part 2: syndromes/patterns (GB/T 16751.2-2021) [Report in Chinese]. https://openstd.samr.gov.cn/bzgk/std/newGbInfo?hcno=C71A9DAD24CB1252F12439D1F045DA6A.

[23] State Administration for Market Regulation, Standardization Administration of China (2023). Clinic terminology of traditional Chinese medical diagnosis and treatment—part 3: therapeutic methods (GB/T 16751.3-2023) [Report in Chinese]. https://openstd.samr.gov.cn/bzgk/std/newGbInfo?hcno=E8BBBAB76E1AF1498C5DA5DFBB2194EC.

[24] Chinese Pharmacopoeia Commission (2020). Pharmacopoeia of the People’s Republic of China [Book in Chinese].

[25] International Classification of Diseases, 11th Revision (ICD-11). World Health Organization.

[26] Hofer M, Obraczka D, Saeedi A, Köpcke H, Rahm E (2023). Construction of knowledge graphs: current state and challenges. SSRN.

[27] Stegeman J, Doriani O’Shee K (2025). How to build a knowledge graph in 7 steps. Neo4j.

[28] Bangor A, Kortum PT, Miller JT (2008). An empirical evaluation of the System Usability Scale. Int J Hum Comput Interact.

[29] Koo TK, Li MY (2016). A guideline of selecting and reporting intraclass correlation coefficients for reliability research. J Chiropr Med.

[30] Fleiss JL (1971). Measuring nominal scale agreement among many raters. Psychol Bull.

[31] National Health Commission of the People’s Republic of China, Ministry of Education, Ministry of Science and Technology, National Administration of Traditional Chinese Medicine (2023). Measures for ethical review of life science and medical research involving human subjects [Report in Chinese]. https://www.gov.cn/zhengce/zhengceku/2023-02/28/content_5743658.htm.

[32] Chen J, Luo M, Chen J (2026). Construction and evaluation of the knowledge graph and large model question-answering system for Jin San Zhen therapy: a tool study for primary care and general practice. Front Med (Lausanne).

[33] Gao Y, Li R, Croxford E (2025). Leveraging medical knowledge graphs into large language models for diagnosis prediction: design and application study. JMIR AI.

[34] Shi J, Ma Y, Liu Y, Shi B (2026). Confident but unsupported: auditing large language models against supplied evidence boundaries in drug-induced liver injury assessment. Front Public Health.

[35] Vasey B, Nagendran M, Campbell B (2022). Reporting guideline for the early-stage clinical evaluation of decision support systems driven by artificial intelligence: DECIDE-AI. Nat Med.

