# Supplementary material for: A Knowledge Graph–Enhanced Question-Answering System for Chinese Patent Medicines in Pediatric Influenza: Development and Evaluation Study
Source: JMIR AI. 2026 Nov 13;5:e101648. doi: 10.2196/101648 (PMC13765779; doi:10.2196/101648)
Supplement: Supplementary file 1 [file ai-v5-e101648-s001.pdf]

CHART Reporting Checklist

Item	CHART recommendation	Status and manuscript location
1a	State that the study is assessing one or more generative AI-driven chatbots for clinical evidence or health advice.	**Reported.** p. 1; Title and Abstract.
1b	Apply a structured format, if applicable.	**Reported.** p. 1; Abstract.
2a	State the scientific background, rationale, and healthcare context for evaluating the generative AI-driven chatbot(s), referencing relevant literature when applicable.	**Reported.** pp. 2, 3; Introduction.
2b	State the aims and research questions including the target audience, intervention, comparator(s), and outcome(s).	**Reported.** pp. 1, 2, 3, 7, 8; Objective; Evaluation of Answer Quality.
3a	State the name and version identifier(s) of the generative AI model(s) and chatbot(s) under evaluation, as well as their date of release or last update.	**Partial.** p. 8; Evaluation of Answer Quality; benchmark file 06.
3b	State whether the generative AI model(s) and chatbot(s) are open-source or closed-source/proprietary.	**Reported.** p. 8; Evaluation of Answer Quality.
4a	State whether the generative AI model was a base model or a novel base model, tuned model, or fine-tuned model.	**Reported.** p. 8; Evaluation of Answer Quality.
4b	If a base model is used, cite its development in sufficient detail to identify the model.	**Partial.** p. 8; Evaluation of Answer Quality.
4c	If a novel base model, tuned model, or fine-tuned model is used, describe the pre- and/or post-implementation/deployment data and parameters.	**Reported.** pp. 3, 4, 5, 8; Corpus and knowledge graph methods; Evaluation of Answer Quality.
5a	Describe the evolution of study prompt development.	**Reported.** p. 8; Evaluation of Answer Quality; benchmark README, Version lineage.
5ai	Describe the sources of prompts.	**Reported.** pp. 7, 8; Evaluation of Answer Quality; benchmark files 01, 04, and 05.
5aii	State the number and characteristics of the individual(s) involved in prompt engineering.	**Reported.** pp. 7, 8; Evaluation of Answer Quality; benchmark file 06.
5aiii	Provide details of any patient and public involvement during prompt engineering.	**Not reported.**
5b	Provide study prompts.	**Reported.** p. 8; Benchmark files 01, 04, 05, and 06.
6a	State route of access to generative AI model.	**Reported.** p. 8; Evaluation of Answer Quality; benchmark file 06.

Item	CHART recommendation	Status and manuscript location
6b	State the date(s) and location(s) of queries for the generative AI-driven chatbot(s) including the day, month, and year as well as city and country.	**Reported.** p. 8; Evaluation of Answer Quality; benchmark file 06.
6c	Describe whether prompts were input into separate chat session(s).	**Reported.** p. 8; Evaluation of Answer Quality; benchmark file 06.
6d	Provide all generative AI-driven chatbot output/responses.	**Reported.** p. 22; Data Availability; benchmark file 02.
7a	Define the ground truth or reference standard used to define successful generative AI-driven chatbot performance.	**Reported.** pp. 7, 8; Evaluation of Answer Quality; benchmark file 01 and README.
7b	Describe the process undertaken for generative AI-driven chatbot performance evaluation.	**Reported.** p. 8; Evaluation of Answer Quality.
7bi	State the number and characteristics of team members involved in performance evaluation.	**Reported.** pp. 8, 9, 18; Evaluation of Answer Quality; Exploratory End-User Usability Evaluation.
7bii	Provide details of any patients and public involvement during the evaluation process.	**Partial.** p. 10; Ethical Considerations.
7biii	State whether evaluators were blinded to the identity of the generative AI-driven chatbot(s) under assessment.	**Reported.** p. 8; Evaluation of Answer Quality; benchmark README.
8	Report how the sample size was determined.	**Partial.** pp. 7, 8, 9; Evaluation of Answer Quality; Exploratory End-User Usability Evaluation.
9a	Describe statistical analysis methods, including any evaluation of reproducibility of generative AI-driven chatbot responses.	**Reported.** p. 9; Statistical Analysis; benchmark README and file 06.
9ai	Report the measures used for performance evaluation.	**Reported.** pp. 8, 9; Evaluation methods.
10a	Report the performance evaluation undertaken including the alignment between generative AI-driven chatbot output and ground truth or reference standard using quantitative or mixed methods approaches as applicable.	**Reported.** pp. 17, 18; Results; Tables 1 and 2; benchmark file 03.
10b	For responses deviating from the ground truth or reference standard, state the nature of the difference(s).	**Partial.** pp. 17, 20; Results and Discussion; benchmark files 01–03.
10c	Report the evaluation for potentially harmful, biased, or misleading responses.	**Partial.** pp. 17, 20, 21; Results and Discussion.
11a	Interpret study findings in the context of relevant evidence.	**Reported.** pp. 19, 20; Discussion.

Item	CHART recommendation	Status and manuscript location
11b	Describe the strengths and limitations of the study.	**Reported.** pp. 8, 21; Limitations; Evaluation of Answer Quality; benchmark README.
11c	Describe the potential implications for practice, education, policy, regulation, and research.	**Reported.** pp. 21, 22; Discussion; Future Directions; Conclusions.
12a	Report any relevant conflicts of interest for all authors.	**Reported.** p. 22; Conflicts of Interest.
12b	Report sources of funding and their role in the conduct and reporting of the study.	**Reported.** p. 22; Funding.
12c	Describe the process undertaken for ethical approval.	**Reported.** p. 10; Ethical Considerations.
12ci	Describe the measures taken to safeguard data privacy of patient health information, as applicable.	**Not applicable.** p. 10; Ethical Considerations.
12cii	State whether permission/licensing was obtained for the use of original, copyrighted data.	**Partial.** pp. 10, 22; Ethical Considerations; Data Availability.
12d	Provide a study protocol.	**Not reported.**
12e	State where study data, code repository, and model parameters can be accessed.	**Partial.** p. 22; Data Availability; benchmark materials.

Source and attribution

CHART Collaborative. Reporting guideline for chatbot health advice studies: the Chatbot Assessment Reporting Tool (CHART) statement. BMJ Med. 2025;4(1):e001632. PMID:40761518 doi:10.1136/bmjmed-2025-001632. https://doi.org/10.1136/bmjmed-2025-001632

Checklist recommendations are reproduced from Table 2 of the CHART statement under the Creative Commons Attribution-NonCommercial 4.0 license (https://creativecommons.org/licenses/by-nc/4.0/). Layout has been adapted and manuscript-specific status, locations, and explanations have been added. This document is prepared for noncommercial scholarly reporting.
